# The many faces of endometriosis

**DOI:** 10.4322/acr.2021.409

**Published:** 2022-11-03

**Authors:** Marcelo Luis Pereira de Souza, Talita Porto da Costa, Nathanael Pinheiro de Freitas, Maiara Ferreira de Souza, Daniel Abensur Athanazio

**Affiliations:** 1 Imagepat, Laboratório de Patologia, Salvador, BA, Brasil; 2 Universidade Federal da Bahia, Hospital Universitário Professor Edgard Santos, Salvador, BA, Brasil

**Keywords:** Endometriosis, Ovary, Pathology

## Abstract

Endometriosis is a common disease; however, unusual findings may cause diagnostic difficulties. We present herein three cases illustrating different morphological appearances of endometriosis: 1) endometriosis with atypical hyperplasia associated with bilateral ovarian carcinoma (mixed clear cell/endometrioid in the left ovary and endometrioid in the right ovary); 2) deep infiltrating endometriosis with intravascular spread, polypoid configuration in peritoneal surfaces, and involvement of a lymph node; and 3) decidualized endometriosis with prominent myxoid/mucinous change and multivacuolated (pseudoxanthoma) cells. Awareness of uncommon morphological manifestations of endometriosis is important to avoid improper consideration of malignancy.

## INTRODUCTION

Endometriosis is a common condition defined by the presence of endometrial tissue outside the endometrium and myometrium. It affects 10-15% of all women during their reproductive years.^1^ In most instances, both endometrial stroma and epithelium are identified, and most cases can be explained by implantation associated with retrograde menstruation. Therefore, the most common endometriosis sites are located near tubal ostia, namely the ovaries, uterine ligaments, rectovaginal septum, cul-de-sac, and pelvic peritoneum.^2^

On rare occasions, endometriosis presents with peculiar features that may raise concerns about malignancy. In the last three years, we have evaluated 244 cases with a final diagnosis of endometriosis. We report, herein, three illustrative cases of progression to carcinoma, intravascular involvement and polypoid growth, and myxoid/mucinous change.

## CASE PRESENTATION

### Case 1

**Figure 1:**
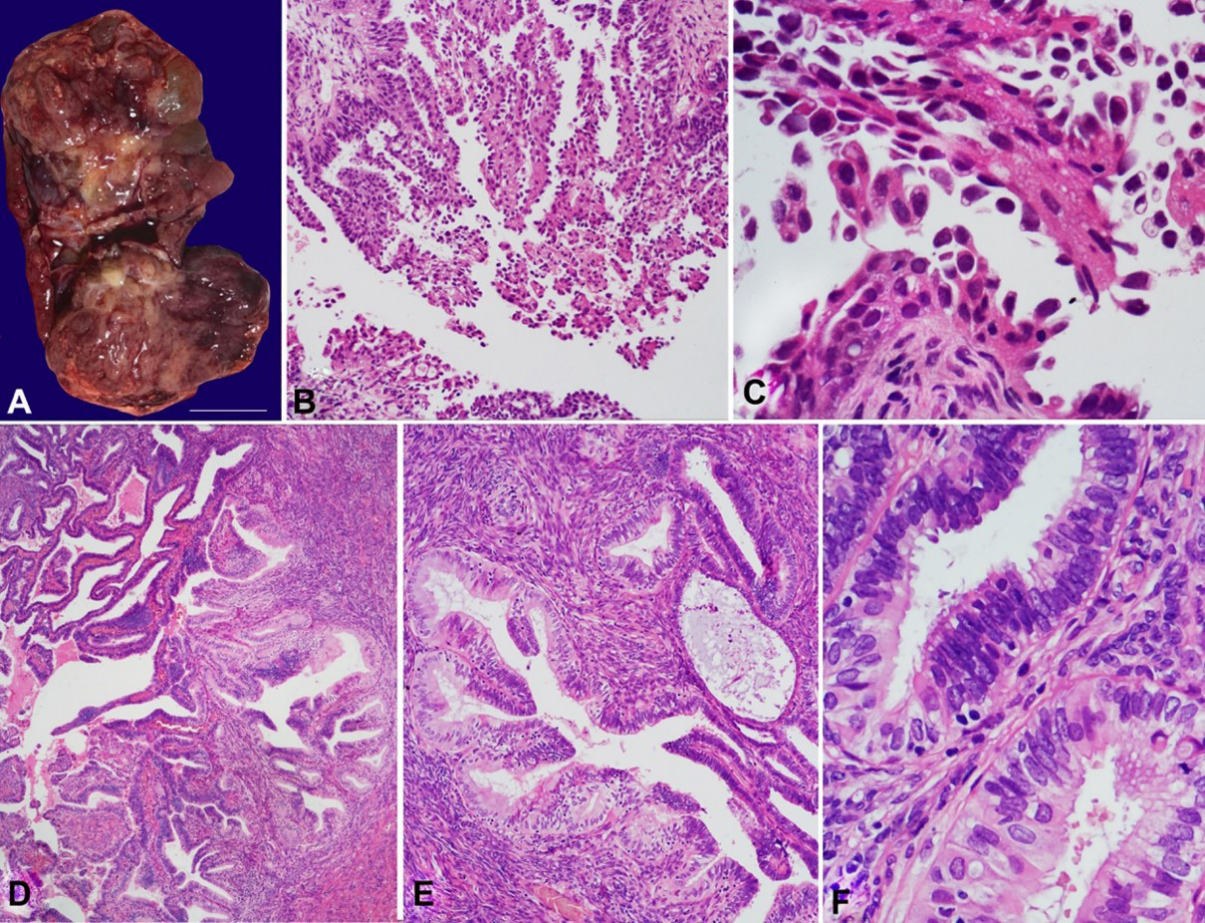
Case 1. Solid and cystic tumor in the left ovary. Cut surface. (**A -** Gross appearance during intraoperative frozen section consultation). Photomicrographs of the clear cell carcinoma component of the mixed carcinoma of the left ovary (**B** - HE, 100×; **C** - HE, 400×). The right ovary showed an endometrioid carcinoma. Adjacent tissues showed atypical endometriosis (**D** and **E** - HE, 100×; F: HE, 400×).

A 53-year-old woman was diagnosed with an ovarian tumor on the basis of imaging studies. She also showed adherences between the uterus, cul-de-sac, and rectum, with an intraoperative impression of endometriosis. The gross (solid and cystic) appearance of the left ovarian tumor is shown in Figure 1A. The final diagnosis was a mixed carcinoma (80% clear cell and 20% endometrioid FIGO grade IIB 2018, pT2b pN0; Figure 1B-C) with foci of carcinomatous infiltration on the mesorectum and peritoneal surfaces (cul-de-sac, bladder dome, and infundibulum). The right ovary showed a 2.5 cm endometrioid carcinoma inside an endometriotic cyst. The peritumoral area exhibited atypical hyperplasia within ovarian endometriosis (Figure 1D-F).

The same criteria for atypical hyperplasia in the endometrium were fulfilled: closely packed glands with epithelium-to-stroma ratio > 3:1; enlarged, rounded, and irregular nuclear contours; coarse and vesicular chromatin; prominent nucleoli; and stratified cells with a loss of polarity. Foci of atypical hyperplasia were also observed in the right fallopian tube. Typical endometriosis was observed in the parametria, mesorectum, and cul-de-sac.

### Case 2

**Figure 2:**
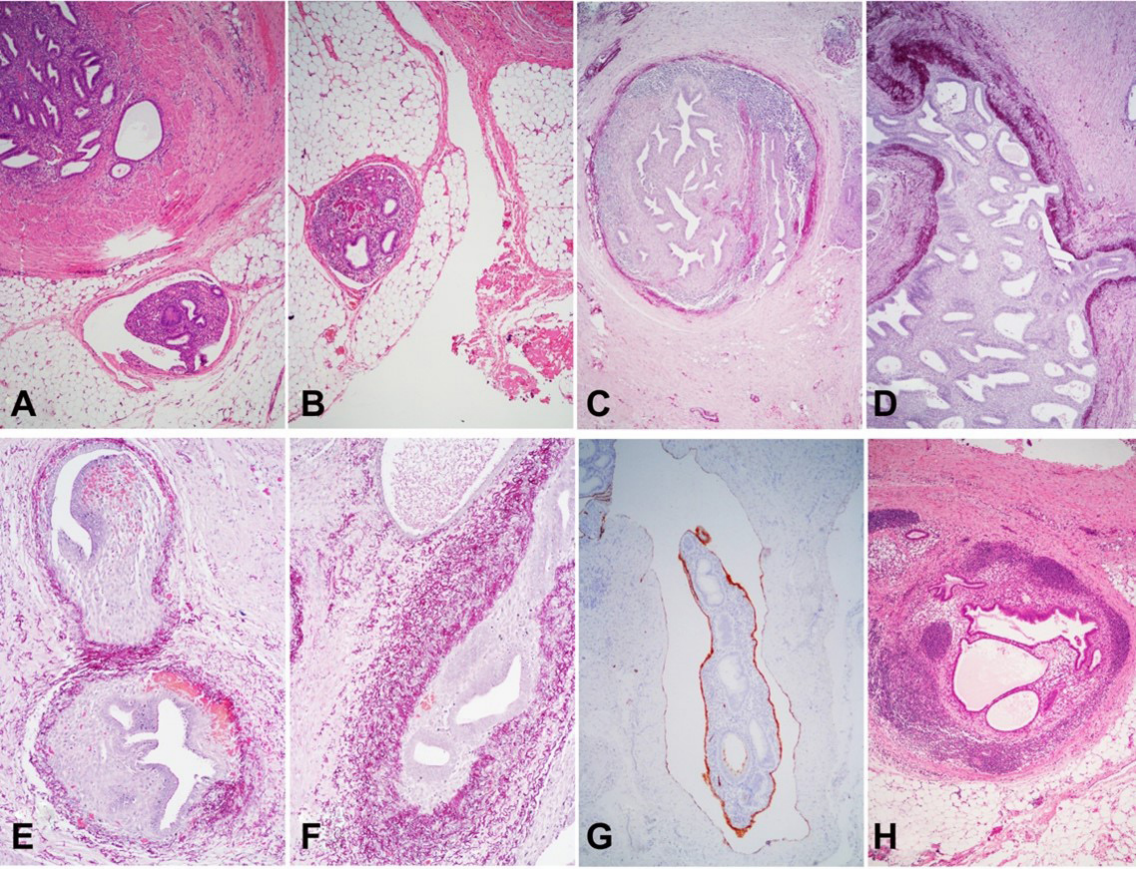
Case 2. Endometriosis within vessels and lymph nodes. Intravascular foci of both endometrial stroma and epithelium (**A** and **B** - HE, 40×). Weigert’s stain highlights the walls of large vessels (**C** and **D** - 40×; **E** and **F** - 100×). Intravascular endometriosis showing D2-40 labeling of both the inner surface of a lymphatic vessel and the external surface of the intravascular mass (**G** - D2-40 immunostain, 40×). An endometriosis focus within a lymph node from the sigmoid mesocolon (**H** - HE, 100×).

A 41-year-old woman underwent abdominal surgery due to multiple adherences between her pelvic organs. The clinical impression and imaging studies suggested malignancy. The intraoperative frozen section consultation and the final diagnosis concluded that this was a case of endometriosis. A florid picture of endometriosis in the left ovary, left fallopian tube, and sigmoid mesocolon was documented. There was no atypia. An extensive intravascular component was noted, and one mesocolic lymph node was involved (Figure 2).

**Figure 3:**
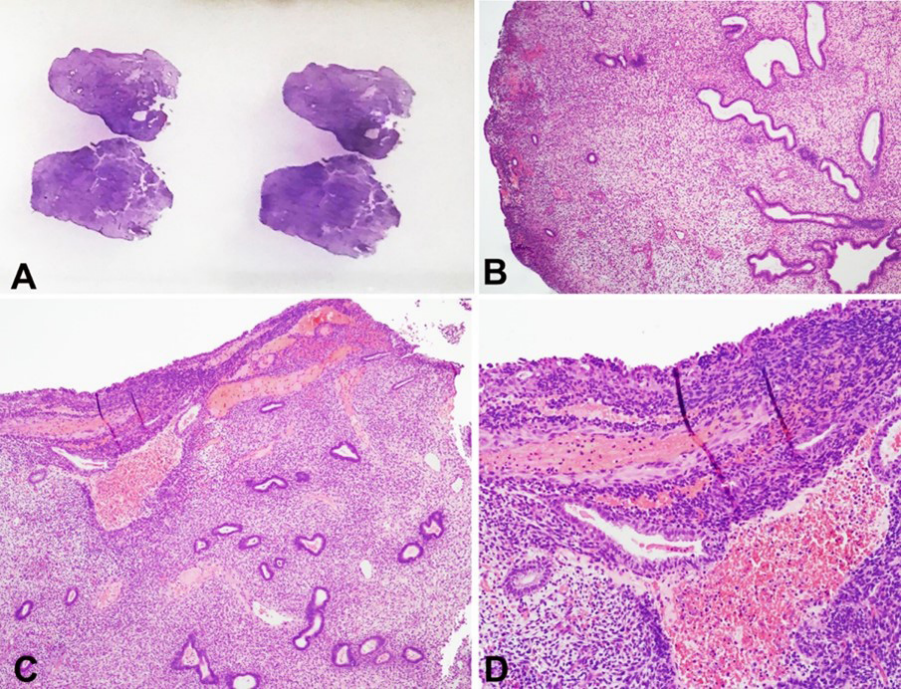
Case 2. Polypoid endometriosis in a specimen removed as an epiploic appendix (**A -** External appearance with HE). The microscopic image is reminiscent of an ordinary endometrial polyp (**B** - HE, 40×; **C** - HE, 40×; **D** - HE, 100×).

A small specimen that was removed and labeled as an epiploic appendix proved to be a polypoid lesion reminiscent of a typical endometrial polyp (Figure 3); this type of lesion has previously been described as polypoid endometriosis.

### Case 3

**Figure 4:**
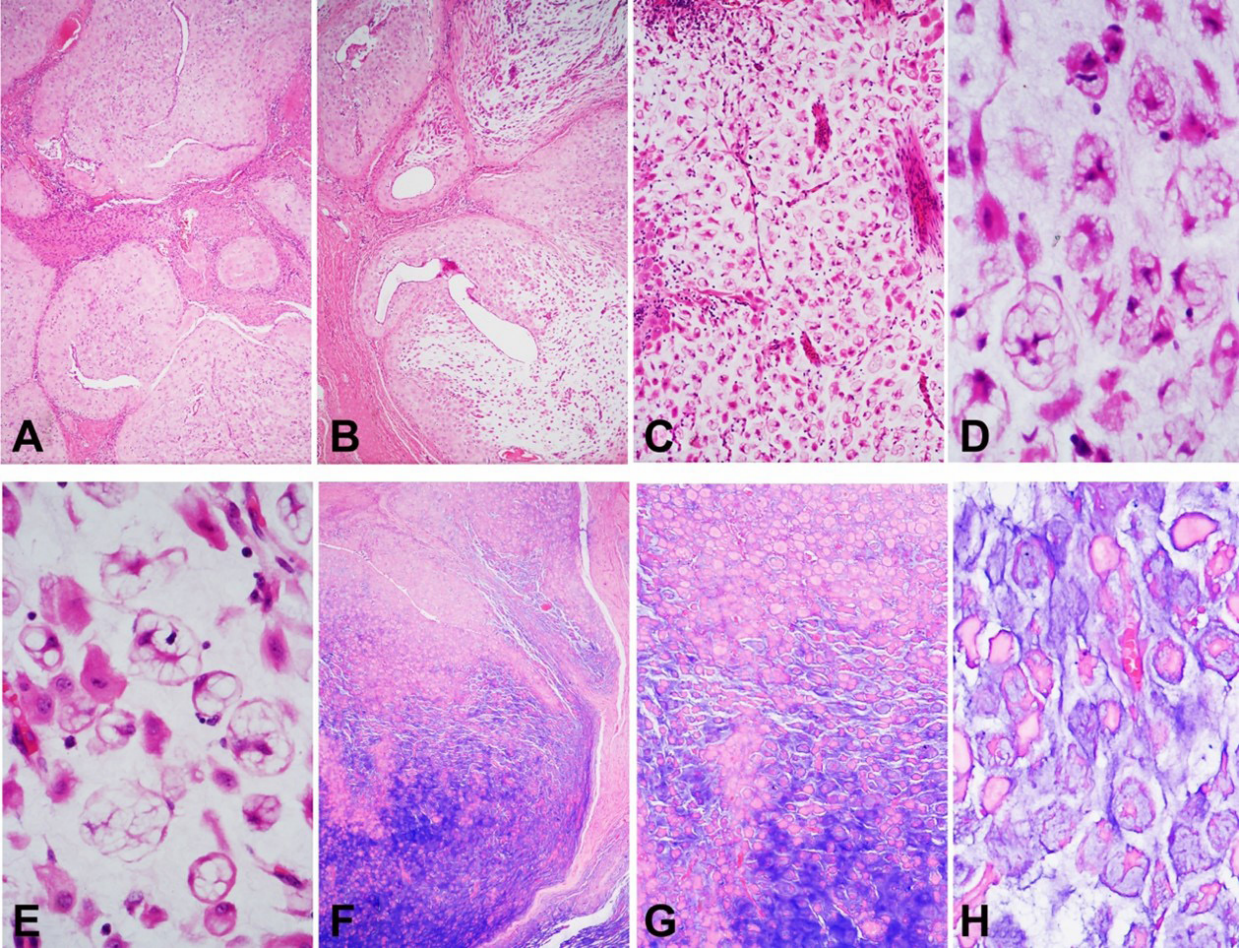
Case 3. Endometriosis in a cesarean section scar. Stromal decidualization and flat/atrophic epithelium (**A** - HE, 40×). Multifocal zones demonstrated a transition from typical areas to myxoid/mucinous changes (**B** - HE, 40×; **C** - HE, 100×). The latter areas showed numerous vacuolated cells (pseudoxanthoma cells) (**D** and **E** - HE, 400×). The Alcian Blue stain highlighted a striking accumulation of mucin (**F** - 40×; **G** - 100×; **H** - 400×).

A 34-year-old woman underwent surgical removal of a lesion with the clinical impression of granular tissue superficial to aponeurosis in a cesarean section scar. When microscopically examined, the endometriosis tissue showed features of the effects of progesterone, such as prominent stromal decidualization and flat/atrophic epithelium (Figure 4A). There were multifocal zones demonstrating a transition from typical areas to myxoid/mucinous changes (Figure 4B-C). The latter areas showed numerous vacuolated cells (pseudoxanthoma cells), and the Alcian Blue stain highlighted a striking accumulation of mucin (Figure 4D-H).

## DISCUSSION

The magnitude of the risk for malignant transformation in endometriosis is difficult to determine. In consecutive specimens removed due to endometriosis, there was concurrent malignancy in 4% of ovaries and 10% of pelvic lesions. Most epidemiological studies estimate that patients with endometriosis have a 1.2 to 1.9 times higher risk of developing ovarian malignancy than those without endometriosis.^3^

The use of the term “atypical endometriosis” is contentious since it has been applied to different situations.^1^ Focal nuclear atypia in endometriotic cysts is a common finding and is probably a reactive process unrelated to malignant transformation. However, this is difficult to evaluate since surgical removal may interrupt disease progression. Some reports have described endometriosis-related malignancies adjacent to endometriosis with foci of nuclear atypia.^4^^,^^5^ Case 1, however, had a much rarer lesion—endometriosis with cytological and architectural atypia—that could qualify (in the endometrium) as atypical hyperplasia/intraepithelial endometroid neoplasia.^1^ When such lesions are observed, they should prompt extensive sampling to exclude a concurrent carcinoma.

Endometriosis with intravascular involvement is a rare observation (Ooi and Valentine 1994; Scolyer et al. 2000).^6^^,^^7^ Its existence supports the metastatic theory of endometriosis development, which may be the most plausible explanation for endometriosis at distant sites, such as the lung, brain, and extremities.^2^ Although considered a benign disease, endometriosis may show features that are sufficient to label other lesions as malignant, such as locally aggressive behavior, risk of recurrence, and intravascular and (probably) metastatic spread. Endometriosis—in particular deep infiltrating endometriosis—shows some genetic aberrations observed in neoplasms. Some authors indeed suggest that deep infiltrating endometriosis would be better defined as a locally aggressive neoplasm that rarely metastasizes.^8^

Another rare manifestation of endometriosis is polypoid growths on serosal surfaces that mimic common endometrial polyps. This lesion type was named polypoid endometriosis by Mostoufizadeh and Scully.^1^^,^^9^ In Case 2, we observed such a lesion in an epiploic appendix.

As we observed in case 2, involvement of lymph nodes seems to be a common occurrence in resected rectal specimens removed due to endometriosis. In a large series, lymph node involvement was observed in 5 out of 35 specimens (26%).^10^

Case 3 showed myxoid/mucinous changes. Myxoid/mucinous changes in endometriosis are rare, but they are well described in decidualized cesarean scars.^11^ Some cases may grossly and microscopically resemble a pseudomyxoma peritonei, with pools of acellular mucin involving peritoneal surfaces.^12^ In small biopsies, this presentation of endometriosis may be difficult to distinguish from pseudomyxoma peritonei with its mucin-rich background and signet ring carcinoma cells. Nevertheless, awareness of this rare presentation of endometriosis is important and may stimulate practitioners to instigate lower thresholds for performing immunostaining. In Case 3, the obvious transition between clear-cut decidualized endometriosis and myxoid/mucin areas allowed recognition of the benign nature of the lesion.

The case presented herein showed no necrotic focus. Another rare morphologic presentation of endometriosis is necrotic pseudoxanthomatous nodules in which a necrotic center is surrounded by histiocytes and/or hyalinized stroma.^13^

In three years, we examined 244 resected specimens that had a final diagnosis of endometriosis. The organs involved were the uterine adnexa (n = 120), peritoneum (n = 30), large bowel (n = 29), appendix (n = 20), abdominal wall (n = 15), bladder (n = 11), skin and subcutaneous tissue (n = 9), parametria (n = 8), ileum (n = 3), vagina (n = 1), and ureter (n = 1).

The frequency of atypical hyperplasia was found to be 2 in 244 (0.8%). A total of 10 cases of endometriosis had coexistent ovarian carcinoma (six endometrioid, two clear cells, and one mixed endometrioid and clear cell) or peritoneal carcinoma (one case of endometrioid adenocarcinoma). Intravascular involvement, polypoid growth, mesocolic lymph node involvement, and myxoid/mucinous changes were observed in one case each (0.4%).

## CONCLUSION

Awareness of uncommon morphological manifestations of endometriosis is important to avoid improper consideration of malignancy.
